# Implementation and challenges to preventing the re-establishment of malaria in China in the COVID-19 era

**DOI:** 10.1186/s12992-022-00858-w

**Published:** 2022-06-21

**Authors:** Guangyu Lu, Yuanyuan Cao, Dongying Zhang, Yuying Zhang, Yuhui Xu, Yan Lu, Qi Chen, Guoding Zhu, Jun Yan, Olaf Müller, Jun Cao

**Affiliations:** 1 School of Public Health, Medical College of Yangzhou University, Yangzhou University, Yangzhou, China; 2 National Health Commission Key Laboratory of Parasitic Disease Control and Prevention, Jiangsu Provincial Key Laboratory On Parasite and Vector Control Technology, Jiangsu Institute of Parasitic Diseases, Wuxi, China; 3 Center for Global Health, School of Public Health, Nanjing Medical University, Nanjing, China; 4 Yangzhou Municipal Center for Disease Control and Prevention, Yangzhou, China; 5 Nanjing Health and Customs Quarantine Office, Nanjing, China; 6 Institute of Global Health, Medical School, Ruprecht-Karls-University, Heidelberg, Germany; 7 Chinese Center for Disease Control and Prevention, Beijing, 102206 China

**Keywords:** Imported malaria, COVID-19, Malaria elimination, Prevention of re-establishment, Integrated health program

## Abstract

**Background:**

The rapid emergence and global spread of COVID-19 have caused substantial global disruptions that have impacted malaria programs worldwide. Innovative strategies to enable countries aiming to eliminate malaria as well as those that are already certified as malaria-free, are needed to address malaria importation in the context of the COVID-19 pandemic. China was certified as malaria-free in 2021 and now aims to prevent the malaria re-establishment. Nonpharmaceutical interventions such as entry screening, quarantining, and health education for individuals returning from international travel during the COVID-19 pandemic present both opportunities and challenges to the management of imported malaria. This study aimed to describe and analyze the operational challenges associated with an integrated surveillance and case management program in which malaria re-establishment prevention measures were incorporated into the COVID-19 program in China.

**Methods:**

After the integration of malaria re-establishment prevention activities into the COVID-19 program for 10 months in Jiangsu Province, China, a focus-group discussion of public health workers working on preventing malaria re-establishment and controlling COVID-19 was held in June 2021, aiming to explore the operational challenges and lessons learned from the integrated approach.

**Results:**

From 01 August 2020 to 31 May 2021, 8,947 overseas travelers with Yangzhou as the final destination underwent 14-day managed quarantine and 14-day home isolation. Of these travelers, 5,562 were from malaria-endemic regions. A total of 26,026 education booklets and materials were distributed to expand malaria-related knowledge. Twenty-two patients with unknown fever were screened for malaria with rapid diagnostic tests, and one patient was confirmed to have imported malaria. The challenges associated with the implementation of the integrated malaria surveillance and case management program include neglect of malaria due to COVID-19, lack of a standard operating procedure for malaria screening, mobility of public health providers, and difficulties in respecting the timeline of the “1–3-7” surveillance strategy.

**Conclusions:**

China’s experience highlights the feasibility of integrated case surveillance and management of existing infectious diseases and new emerging infections. It also demonstrates the importance of a sound public health infrastructure with adequate, trained field staff for screening, testing, contact tracing, and providing health education, all of which are crucial for the success of both malaria re-establishment prevention program and the effective control of COVID-19.

**Supplementary Information:**

The online version contains supplementary material available at 10.1186/s12992-022-00858-w.

## Background

Despite major progress in the fight against malaria, this mosquito-borne disease remains a major public health problem, with approximately 230 million cases of malaria and 430 000 malaria-related deaths reported globally in 2020 [1]. Since 2020, the rapid emergence and spread of coronavirus disease 2019 (COVID-19) worldwide has been caused substantial global disruptions that are impacting malaria programs [2]. The malaria burden may surge because of the direct and indirect effects of the pandemic on malaria-endemic countries [1, 3]. However, in countries working to eliminate malaria or countries that are malaria-free, prevention of the re-establishment of malaria may also be disrupted due to the COVID-19 pandemic [3, 4]. Thus, the COVID-19 pandemic has the potential to hinder ongoing malaria programs not only in endemic areas, but also in countries with recent malaria elimination [4].

Many countries have achieved malaria elimination in recent years, and there has been an increasing focus on the prevention of malaria re-establishment (POR) [5–7]. In malaria-free countries, POR refers to the prevention of malaria outbreaks/epidemics and the prevention of re-establishment from imported malaria [8]. There are commonalities between POR and COVID-19 programs, for example, the success of both program types may be hindered by imported infections and requires stringent case surveillance, testing and follow-up [4]. Furthermore, both programs require collaboration among many diverse sectors in addition to the health care sector [4]. Therefore, in the context of the COVID-19 pandemic, integrating malaria prevention activities into COVID-19 control programs is of vital importance to maintain POR programs in countries certified as "malaria-free" or in countries that will be certified in the near future.

In China, malaria was one of the most serious public health problems, with more than 30 million malaria cases annually before 1949 [9]. However, the disease burden has sharply declined since the implementation of an integrated malaria control strategy. and the incidence of malaria decreased gradually by 2000, with only 20 cases per one million residents [9, 10]. Although malaria resurged in central China between 2001 and 2006 [11, 12], intensified control efforts since 2007 resulted in a further decrease in the incidence, with less than 6 cases per one million residents in 2010 [13]. The National Malaria Elimination Program (NMEP) was launched in China in May 2010, aiming at eliminate malaria by 2020 [14]. In 2021, China was certified as a malaria-free country and now aims to prevent the re-establishment of malaria [15].

In response to the outbreak of COVID-19 in Wuhan at the beginning of 2020, Chinese authorities reacted rapidly and have implemented large-scale public health measures. Although a number of outbreaks of COVID-19 have occurred in several cities in China during the past year, they have all been controlled through intense public health measures [16–19]. China adopted a strict suppression strategy to reduce the incidence; under the suppression strategy, nearly all infections are identified and controlled through rapid testing, backward and forward tracking of infected people, and contact tracing [18].

With the gradual loosening of border restrictions in many countries, returnees from malaria-endemic countries are at risk of for importing both, malaria and COVID-19 infections. Here, we described how the POR program for malaria has been integrated into the well-functioning COVID-19 surveillance and response program of Jiangsu Province, China.

## Methods

### Study area

**Fig. 1:**
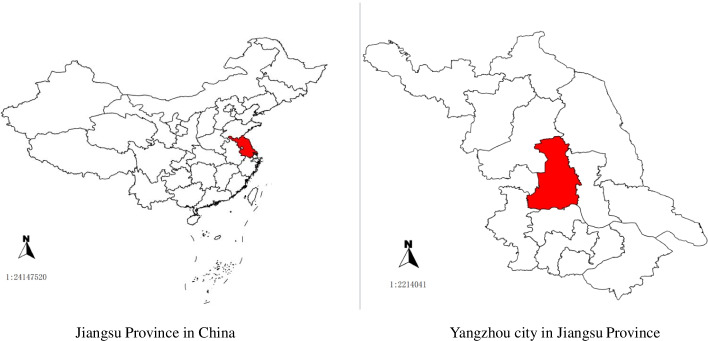
Yangzhou city in Jiangsu Province and China

Yangzhou is a prefecture-level city in central Jiangsu Province in eastern China (Fig. 1 A). Yangzhou city had an estimated total population of 4.55 million people in 2020 (Metro: 6,592 km^2^) and borders the provincial capital of Nanjing [20]. In 2010, Yangzhou’s gross domestic product (GDP) reached 220.8 billion yuan ($35.73 billion) [21]. There are 5 functioning subdistricts (Hanjing county-level city, Yizhen county-level city, Jiangdu District, Guangling District, and Baoying County). There are 3 tertiary hospitals, 76 secondary and primary hospitals, and 70 community health centers (CHCs) in Yangzhou city.

**Fig. 2:**
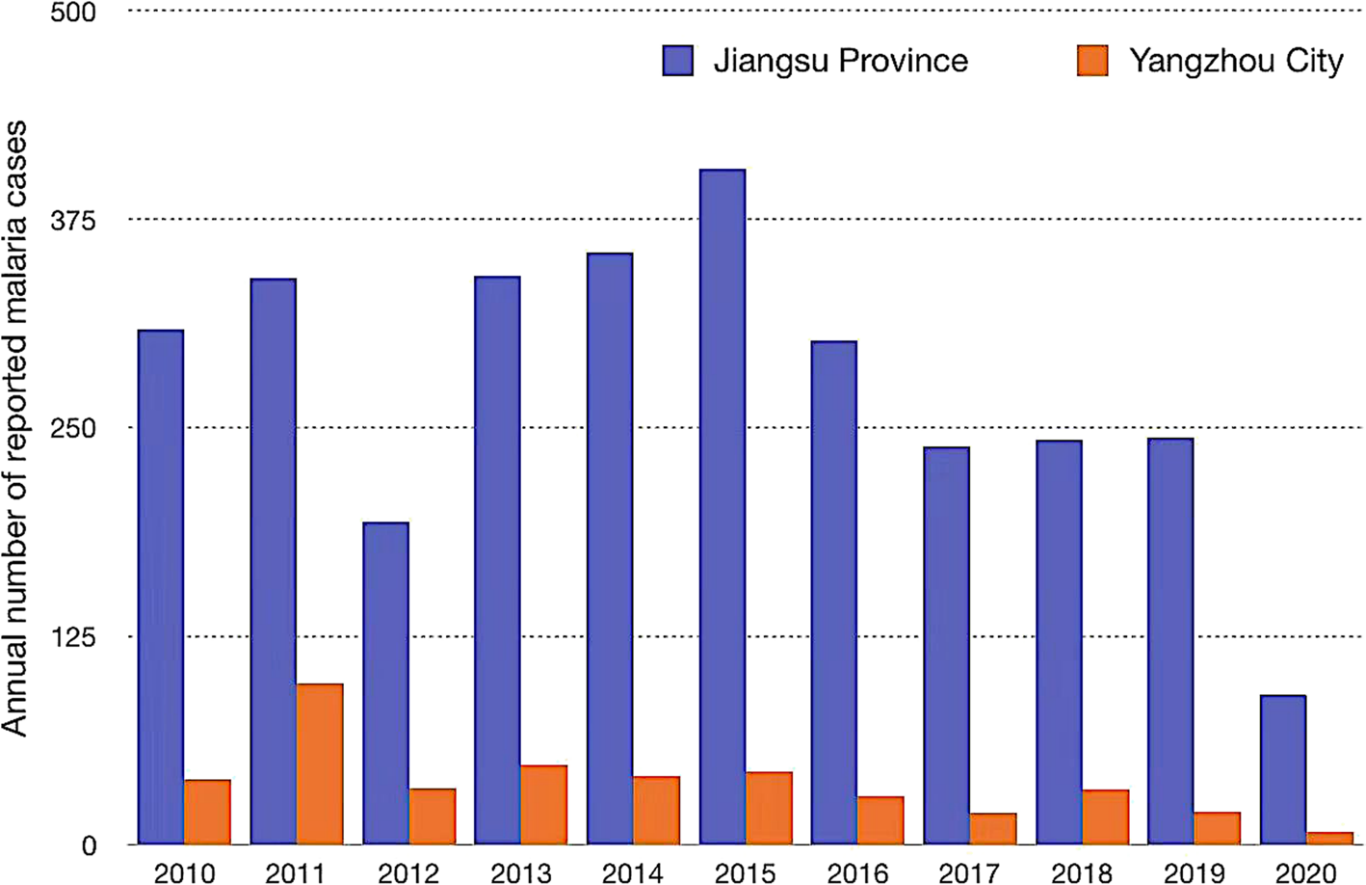
Reported number of malaria cases in Jiangsu Province and Yangzhou City, 2010–2020

Since 2012, no cases of locally transmitted malaria have been reported in Jiangsu Province. However, the number of cases of imported malaria in Jiangsu Province was among the highest in China, with the yearly number ranging from 198 to 405 from 2012–2019 (Fig. 2). Moreover, there are a large number of migrant laborers in Yangzhou, with an estimated 5,805 overseas international laborers working in the city annually [22].

### POR program for malaria and COVID-19

**Fig. 3:**
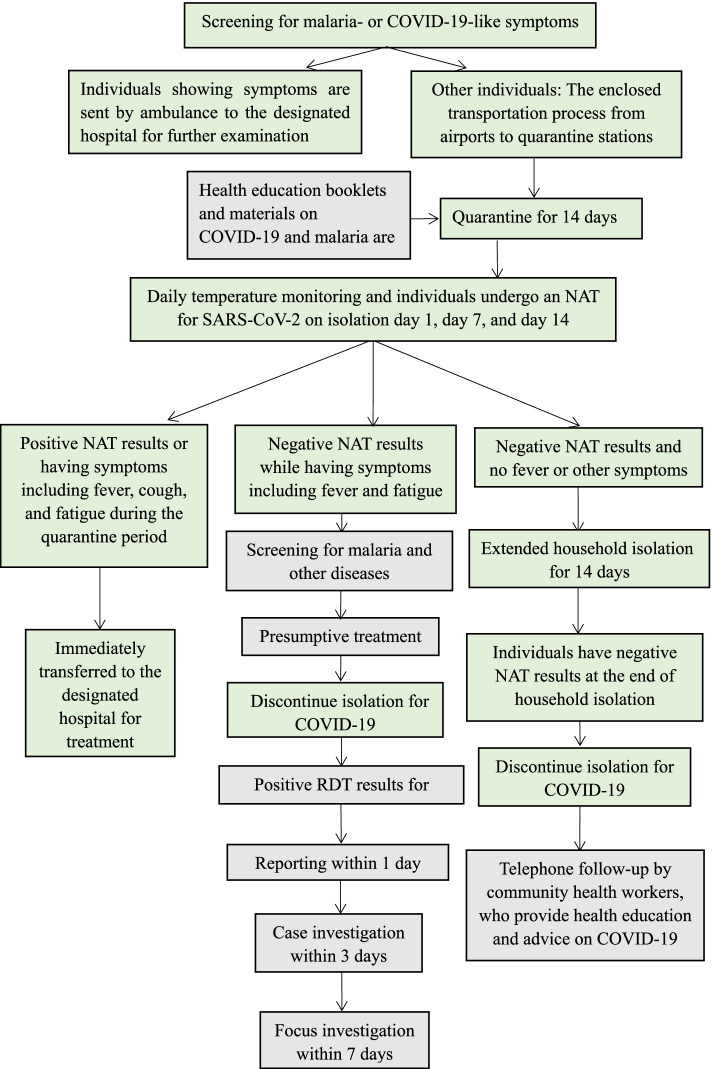
The close-loop management of overseas travelers

The working flowchart of the integrated approach is illustrated in Fig. 3.

### Before arrival

In the COVID-19 program, before overseas travelers arrive in Yangzhou (as their destination), responsible health staff in charge of transportation inform the local City Center for Diseases Control and Prevention (Yangzhou CDC) of all details of the returning travelers, including which countries they are arriving from and the location of their quarantine stations.

### Airport education

Leaflets and posters on the POR of COVID-19 and malaria are available on the airplane and at immigration desks in airports. Upon arrival at the airport, the returnee will receives a designed luggage card on which online social media QR codes are printed (Supplementary material, Figure S1). Information on malaria-endemic countries, information on malaria prevention and treatment, and post-travel health consultation hotline numbers can be accessed at any time through a social media app by scanning the QR codes on a mobile phone.

### Closed-loop management strategy

To prevent imported infections of COVID-19, China has implemented a closed-loop management strategy, with the cooperation of customs, immigration, health bureaus and other departments, that includes nucleic acid testing (NAT) for severe acute respiratory syndrome coronavirus 2 (SARS-CoV-2) and 14 days of medical observation for individuals with a history of overseas travel [23]. For international travelers with Shanghai and three neighboring provinces (Jiangsu, Zhejiang or Anhui provinces) as their final destinations, the closed-loop management strategy consists of three loops [24]. The first is activated upon the a passenger’s arrival at the airport. People showing symptoms are sent by ambulance to a hospital for further examination. The second loop refers to the enclosed transportation process, in which passengers without symptoms are sent from airports by specially assigned vehicles to designated quarantine locations in their destination cities. The last is the management of quarantine stations, which are staffed by disease control professionals as well as medical and service personnel from local clinics and communities (Supplementary material Figure S2) [24].

### Screening for malaria during quarantine and home isolation for COVID-19

Upon the activation of the second loop, overseas travelers are immediately dispatched to quarantine stations for 14 days followed by supervised self-quarantine in their homes for an additional 14 days. During the 28-day quarantine period, travelers are required to undergo NAT for SARS-CoV-2 on day 1, day 7, day 14 and day 28. If a traveler is returning from a malaria-endemic country, the regional malaria program manager is informed by the health staff of the entry city (for example, Shanghai) and ensures that the traveler is recorded and further screened for malaria by rapid diagnostic tests (RDTs) if a fever of unknown origin occurs.

### Health education during the quarantine period

Leaflets and brochures on the signs and symptoms of malaria as well as free treatment availability, free-of-charge RDTs, blood testing after returning from a malarious country, and hotline numbers are available in the quarantine hotel rooms.

### Postquarantine follow-up

**Fig. 4:**
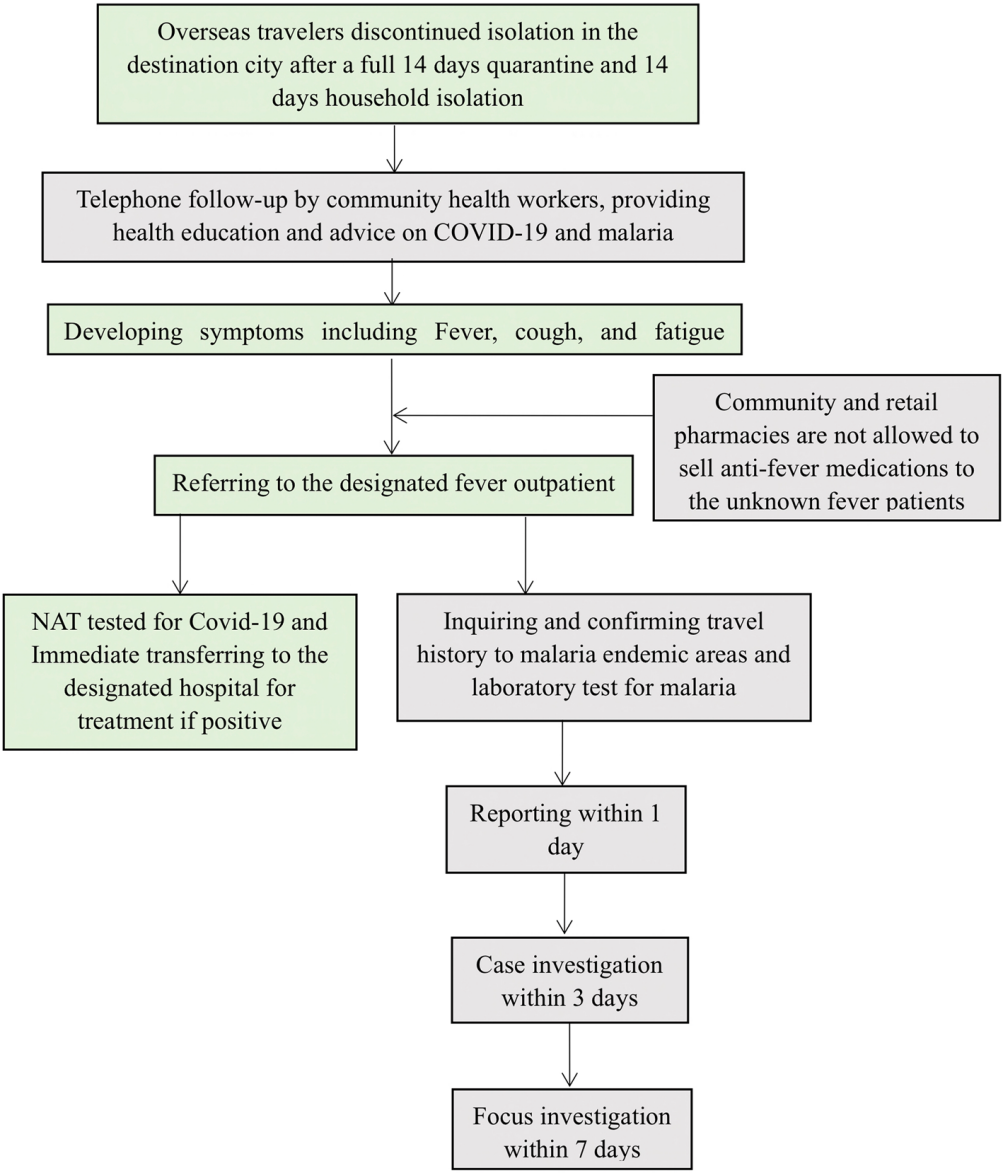
Flowchart of the working scheme for the integrated POR program for malaria and COVID-19 after discontinuing isolation in Jiangsu Province, China

To prevent deaths attributed to malaria that result from delayed care seeking, at the end of the quarantine period, program staff reaffirm messaging regarding prompt care seeking through follow-up telephone calls and household visits, while following all local personal protection and physical distancing guidelines established by facilities and local authorities (Fig. 4).

### “1–3-7” malaria surveillance and response

If a malaria case is suspected, China’s “1–3–7” malaria surveillance and response protocol is followed. China’s “1–3–7” approach defines targets to guide and monitor case reporting, investigation, and response; the 1–3–7 approach requires the reporting of malaria cases within one day, confirmation and investigation within three days, and an appropriate public health response to prevent further transmission within seven days [25].

### The implementation of the program

The integrated POR program for malaria and COVID-19 programs has been implemented in Yangzhou since mid-2020. The implementation details of the integrated malaria surveillance and case management program are reported on a monthly basis to the Yangzhou CDC and validated by the Jiangsu Institute of Parasitic Diseases, Wuxi, Jiangsu Province. For this paper, overseas travelers who quarantined for COVID-19, were screened for malaria, and were educated about malaria from 1 August 2020 to 31 May 2021 were analyzed.

### Focus group discussion (FGD)

To gain a wide range of views and to stimulate reflection on challenges associated with the integrated malaria surveillance and case management program, a qualitative exploratory focus group methodology was adopted. The focus group discussion was incorporated into a provincial-level meeting that was specifically organized to share experiences and lessons learned and discuss challenges during the implementation of the integrated approach. Public health workers from the Yangzhou local CDCs (*n* = 5) and malaria program managers from the Jiangsu Provincial Institute of Parasitic Diseases (*n* = 3) were enrolled for the FGD. We selected and invited participants via purposive sampling to ensure that they all fully worked with the malaria POR program. Moreover, the 5 CDC staff were partly involved in the COVID-19 program. The discussion was moderated by an experienced local public health administrator with many years of experience in malaria control and elimination. The moderator used semistructured scripts with open-ended questions and prompts to guide the discussions, which primarily centred on identifying and discussing the challenges and lessons learned during the implementation of the integrated malaria surveillance and case management program (Supplementary material table 1). The discussion lasted for approximately two hours.

We performed a qualitative analysis through a directed content analysis of the FGD transcripts assisted by the qualitative analysis software MAXQDA 12. For this purpose, the transcripts of the discussion were recorded by the main researcher (GYL), transcribed literally (GYL and YYC), and reviewed for accuracy (JC). The inductive data analysis process followed Clarke and Braun’s seven steps, including transcription, reading and familiarization, coding searching for themes, reviewing themes, defining, and naming themes and finalizing the analysis [26]. Six research team members who were experts in the prevention of malaria reintroduction, and four of the research team members had community-level experience working with both malaria and COVID-19.

### Ethics

Before the start of the focus group discussion, the purpose of the meeting was introduced, and consent for participation was obtained from all the participants. Data were anonymized prior to statistical analysis. The study was registered as part of a nationally funded research project with the aim of improving the case management of patients with imported malaria (registration no. 71904165). Ethical approvals for the study were received from the Ethics Committee of Medical College of Yangzhou University.

## Results

### Implementation of the integrated POR program for malaria and COVID-19

**Fig. 5:**
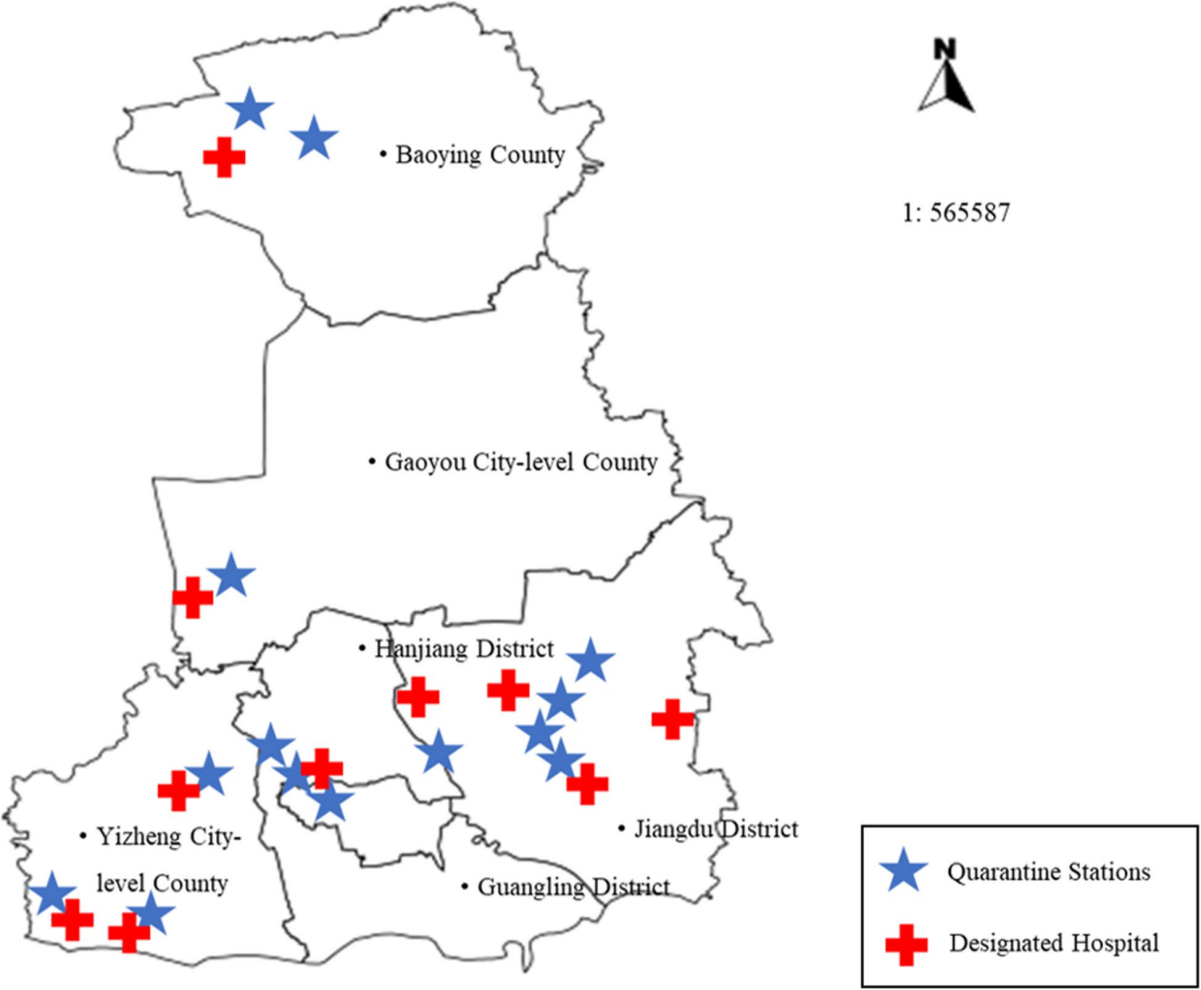
The distribution of the 13 quarantine stations and 10 designated hospitals in Yangzhou city

According to the population densities in the subdistricts, 13 quarantine stations were established in Yangzhou. Each quarantine station had a designated hospital nearby, which was prepared to treat potential COVID-19 cases (Fig. 5). From 1 August 2020 to 31 May 2021, 8,947 overseas travelers to Yangzhou city were quarantined at the 13 quarantine stations (Supplementary material Table 2). Of these travelers, 5,562 returned from highly malaria-endemic areas, mainly countries in sub-Saharan Africa (SSA). During the quarantine period, 26,026 health education materials on malaria were distributed to 8,947 returning travelers. On 30 March 2021, a case of imported malaria was detected at a quarantine station in a migrant worker returning from the Democratic Republic of the Congo. He developed fever on 10 April 2021 (on quarantine day 10). The NAT was negative for SARS-CoV-2; thus, malaria was suspected. Treatment with artemisinin-based combination therapy (artemisinin and piperaquine tablets) was immediately provided. Upon completion of the 14-day isolation period at the quarantine station on 14 April, the RDT for malaria was positive. This patient with imported malaria was reported to the Chinese Information System for Disease Control and Prevention (CISDCP), an internet-based reporting system. An epidemiological survey was carried out and reported in the system on the same day. Malaria species classification by microscopy was performed by local CDC staff on 15 April 2021 and later confirmed as *Plasmodium falciparum* by PCR. The local CDC staff provided health education materials on malaria to the patient and his family and neighbors on the third day after the case was reported.

### Operational challenges associated with the integrated POR program

In general, the public health providers working in local CDCs evaluated the integration of malaria surveillance and case management with the current COVID-19 program as successful and efficient. However, five themes regarding operational challenges that were related to the integrated POR program were identified.

#### Neglect of malaria due to COVID-19

The health workers were concerned about maintaining malaria activities under the current COVID-19 program because they felt that the populace and public health providers may give more priority to COVID-19 and thus neglect the threat of malaria. Health workers may think it is not necessary to put efforts into preventing malaria because it has already been eliminated and is rarely encountered.

“*It is without doubt that the prevention and control of COVID-19 is the priority. Malaria may be neglected after the achievement of the elimination goal. The situation is even worse in the context of the COVID-19 pandemic.*” (FGD participant).

#### Lack of a standard operating procedure for malaria screening

The local CDC staff members were concerned about the lack of a standard operating procedure (SOP) for malaria screening in overseas travelers in quarantine stations. Malaria is suspected in any patient with a febrile illness who has recently returned from a malaria-endemic country. Before the COVID-19 pandemic, blood smears were obtained from patients with suspected or RDT confirmed malaria by local CDC staff and sent to the Provincial Institute for microscopic verification. A blood-spot filter paper sample was also delivered to the reference laboratories in the Provincial Institute for molecular verification with PCR. However, in the context of the COVID-19 pandemic, there were uncertainties about whether malaria screening should be performed during or upon completion of the quarantine period for COVID-19. Should a blood smear be obtained for malaria verification during the quarantine period? Who should be in charge of taking a blood smear during the quarantine period? Is an RDT alone adequate for screening at this stage? These questions indicated an urgent need to develop an SOP for malaria screening, particularly during the quarantine period.

“*The health staff in the quarantine stations are mainly in charge of monitoring temperature or performing testing for COVID-19, and they may not be in charge of malaria-related activities or may have limited experience in screening for malaria. Moreover, considering that returning travelers are at increased risk of COVID-19, is it safe for health staff to take blood samples during the quarantine period? I am not sure. I am also not sure whether it is safe to transfer the blood smear.*” (FGD participant).

#### Delay of medical care for malaria patients

The health workers generally expressed concern that due to COVID-19, malaria patients are at risk of being delayed treatment. Designated infectious disease hospitals in which COVID-19 patients are hospitalized are quarantined and cannot receive patients with other infectious diseases, including malaria.

“*The City Third People’s Hospital is designated to receive and treat malaria patients. The doctors specialized in infectious diseases. However, if COVID-19 patients are hospitalized in this hospital, the hospital will not receive any malaria patients. In this case, we need to coordinate with other hospitals to receive malaria patients.*” (FGD participant).

#### Mobility of public health providers

In the COVID-19 program, the quarantine stations are staffed with medical and public health providers from local CDCs and community health centers. They usually take turns working in the quarantine stations. Thus, public health providers in quarantine stations have great mobility. They may not be well trained on malaria programs.

“*The COVID-19 program health workers at quarantine stations usually have great mobility. Therefore, we always need to train and remind these health workers about the malaria program, for example, when to suspect a case of malaria and to provide health education regarding malaria. However, their extensive mobility makes it difficult to maintain consistency in the POR program.*”

#### Difficulties in respecting the timeline of the “1–3–7” surveillance strategy

The health workers mentioned that due to the COVID-19 pandemic, the achievement of China’s “1–3–7” strategy is challenging. In particular, performing species classification within 3 days is difficult. A microscopic test to classify species is carried out only after the completion of the 14-day quarantine period and confirmed negativity for COVID-19 is confirmed.

“*Reporting the case within 1 day is fine; however, sometimes it is difficult to classify the malaria species within 3 days. If the patient is in a quarantine station, we are not sure of the COVID-19 infection status of the patient. In these cases, we had to wait until the end of the full quarantine period to take a blood smear for species classification.*”

## Discussion

This study described the integration of the POR program for malaria into the COVID-19 program in Jiangsu Province, China, which could be as a reference for closed loop management. Integrated surveillance and case management are promising strategies that can be used to advance elimination goals and maintain malaria elimination success in the context of the COVID-19 pandemic. The operational challenges of the integrated POR program included the lack of an SOP for malaria screening during quarantine, medical care delays for malaria patients, the extensive mobility of public health providers, difficulties in achieving the timeline targets of China’s “1–3–7” strategy, and the neglect of malaria control activities due to COVID-19 priorities. These challenges need to be considered during program efforts to achieve malaria elimination or for the POR of malaria.

### Implementation of the integrated POR program for malaria and COVID-19

#### Integrated malaria surveillance during COVID-19 quarantine

Countries with open borders are implementing health screening and quarantine measures that are essential for the identification of potential COVID-19 cases and the prevention of further transmission in communities [27]. The quarantine period for COVID-19 presents an opportunity to respond to other imported infectious diseases to prevent unnecessary morbidity and deaths, particularly in countries with good health systems and available diagnostic capacities. Our study suggests that rapid diagnostic testing by health care workers for malaria during COVID-19 screening is feasible and effective. Malaria shares some highly recognizable symptoms with COVID-19, such as fever, difficulty breathing, fatigue and headache with acute onset [28]; therefore, it is important to differentiate malaria from COVID-19 through laboratory investigations [29]. Symptoms of malaria usually develop 10–15 days after being bitten by an infected *Anopheles* mosquito; however, shorter periods have been observed in patients with *P. falciparum* infection, and longer periods have been observed in patients with *P. malariae*, *P. ovale*, and *P. vivax* infection [30]. This implies that screening for malaria during the 14-day quarantine period, which was adopted by many countries during the COVID-19 pandemic, may be effective for the detection of *P. falciparum* infection but not always for nonfalciparum infections. Although the majority of patients with imported malaria in China are migrant workers returning from SSA who are infected with *P. falciparum* [31, 32], proper attention should be given to the early recognition and diagnosis of non-falciparum malaria in a nonendemic region. In some cases, COVID-19 coinfection may compound this challenge due to overlapping symptoms [33]. Therefore, returning travelers who present with symptoms of fever, headache, and myalgia should be investigated for malaria infection during the COVID-19 quarantine period and postquarantine period.

#### Integrated health service delivery for fever patients

The misdiagnosis and inappropriate treatment of COVID-19 and malaria infections pose an immediate health threat to the individual and have public health consequences for the community. A challenge with regard to misdiagnosis in countries working toward malaria elimination is that people with fever may preferentially undergo testing for COVID-19 and be sent home due to a negative result [29]. Another scenario is that patients may have malaria and COVID-19 coinfection, but the diagnosis and treatment of one may lead to overlooking the other [28, 34]. Therefore, the provision of appropriate and clear medical tests for fever patients is crucial, as the following clinical manifestations of COVID-19 and malaria largely overlap: Fever, headache, joint pain, respiratory symptoms, and general weakness [3]. In our study, patients in the community who developed fever were directed to designated fever clinics with medical capacity for the diagnosis of COVID-19 and malaria. Private clinics, pharmacies and local drug sellers are not allowed to sell antipyretics to patients with fever of unknown origin. This strategy largely decreases the possibility that overseas travelers with malaria or COVID-19 will self-treat with antipyretics, which may delay health care consultation, diagnosis and appropriate treatment. Integrating medical consultations and tests for fever patients with suspected malaria in designated COVID-19 fever clinics is crucial for the accurate diagnosis and effective treatment of malaria.

#### Integrated health education

Maintaining community awareness and community participation are important in malaria POR programs. To mitigate the potential for the transmission of COVID-19, intensive health education about prevention measures, such as good hygiene practices, cough etiquette, and social distancing, is needed. Therefore, there is great potential to integrate community education and health promotion regarding malaria into COVID-19 activities. Integrated community education saves time and energy and reinforces messaging regarding malaria prevention, diagnosis and treatment, particularly for international travelers returning from malaria-endemic areas. For example, booklets about malaria could be placed in quarantine stations for individuals in mandatory quarantine for COVID-19. Moreover, during the postquarantine telephone follow-up, health advice and education on malaria and COVID-19 could be provided together. Integrated health education and health promotion will be beneficial for both programs.

#### Integrated safety training for public health providers

During the COVID-19 response, many community health care workers were in charge of both the malaria POR program and COVID-19 programs; therefore, integrating the professional training of health workers to ensure safe working environments and properly resourced facilities is essential. Community-based malaria workers are in close proximity to febrile patients and are at high risk of contracting COVID-19. In contrast with Ebola, for which any exposure to blood is an important risk factor for transmission, exposure to blood is not a recognized risk factor for COVID-19 infection [35]. Health worker-patient encounters should be managed using existing preventive measures (e.g., mask and glove wearing, handwashing and glove wearing) to reduce COVID-19 transmission.

### Operational challenges associated with the malaria POR program in the context of COVID-19

#### Potential disruptions to POR programs

In the context of COVID-19, funds and personnel may be reassigned from malaria programs and other programs to COVID-19 response efforts; thus, malaria control and elimination efforts may be hindered or neglected as great efforts are made to control the COVID-19 pandemic [36]. Countries that have successfully eliminated malaria or are approaching elimination face similar risks of resurgence if the current malaria programs are substantially disrupted by the COVID-19 pandemic [36, 37]. Similar phenomena were reported during the Ebola virus outbreak in West Africa; deaths from malaria, HIV and tuberculosis surged, and malaria deaths during the Ebola outbreak exceeded the number of deaths from the Ebola virus in parts of West Africa [38–40]. Contributing factors included deaths of health care workers, overwhelmed health facilities and fear of contracting the disease at health service centers [38, 41]. The lessons learned from the Ebola crisis indicate that stakeholders in health sectors should prevent the disruption of malaria programs during the planning and implementation of future disease control programs, such as the COVID-19 program. On the other hand, integrating the malaria POR program into the COVID-19 program based on commonalities in testing, contact tracing, and education provides an innovative way to sustain malaria POR program during the COVID-19 pandemic.

#### Lack of an SOP for malaria screening

Screening for malaria during quarantine for COVID-19 is an innovative strategy for early detection; however, the questions of when to screen, how to screen and who should perform the screening remain unanswered. The absence of a malaria screening SOP may confuse health workers in quarantine stations who work on malaria activities. For example, in China, travelers in quarantine undergo an RDT for malaria, because of the widespread safety concerns among health workers about their risk of exposure to COVID-19 while obtaining blood smears. The sensitivity of the RDT is affected by low parasitic densities; thus, the limited capacity of an RDT to screen asymptomatic cases is a challenge in malaria screening programs [42]. Therefore more sensitive field diagnostics are needed [43]. Moreover, the experiences of health staff in China indicated that the importance of the awareness of suspicion of malaria during the COVID-19 context, particularly during the period of the closed loop management. Moreover, in the postquarantine period, travelers in the community are mainly followed by telephone to encourage proper care seeking after symptom onset, while in Sri Lanka, travelers are required to undergo blood screening for malaria parasites by microscopy 3, 6 and 12 months after returning to their home, and they are advised to report for malaria testing if they develop fever [4]. The experiences of health staff in China indicated that following up on the health status of the returning travelers and providing proper health education, even only by telephone, make sense. These results further suggest the need for the development of context-based SOPs for malaria screening.

#### Logistic difficulties

Maintaining malaria elimination relies on early detection, accurate reporting, and proper response to potential infections, but malaria programs are at particular risk of being scaled back for logistical reasons associated with COVID-19, putting communities at risk [44]. Our study identified that the completion of malaria case investigations within three days after reporting was threatened by the fact that blood smears were not obtained until the patients were confirmed to be negative for COVID-19. If a patient was suspected to have malaria and tested positive using an RDT, case classification by blood smear was performed only after the completion of the full quarantine period. Moreover, in some COVID-19-endemic countries, travel restrictions may make it difficult for some health workers to travel to their workplaces, while others may become infected with COVID-19, leading to a much-reduced health system capacity [3, 44–46]. These complex contexts compromise the provision of health care resources and surveillance for malaria and may threaten elimination efforts.

#### Delayed care seeking

As observed during the Ebola outbreak, fear and distrust of the health care system can negatively impact care seeking [47, 48]. Furthermore, early in the COVID-19 pandemic, local media encouraged individuals experiencing mild fever or nonsevere symptoms to stay home. Malaria programs should anticipate that individualsand their families may be reluctant to seek care due to the risk of potential COVID-19 exposure. Therefore, innovative targeted care delivery approaches (such as online remote medical consultation or virtual doctor visits) and communication efforts encouraging high-risk groups to promptly seek medical care in line with local policies are important.

Since the beginning of the COVID-19 epidemic in China, very efficient networking and highly organized and well-coordinated operations have taken place between units of the Ministry of Health and other sectors, including the Ministries of Defense, Airport and Aviation, and Foreign Affairs and Police Departments [49, 50]. This multisector cooperation has been critical to the success of both programs, in which the military and the police have played a major role. For example, contact tracing and quarantine practices require substantial effort by health care workers and impose a heavy burden on the health system; in many countries, this burden was shared equally among the Ministry of Health, police department and the military, whose expertise and resources were rapidly mobilized [4, 51]. Certainly, this strengthened multisector cooperation will benefit not only the malaria POR programs for these two diseases but also the public health preparedness capacity for future emerging infectious diseases.

## Conclusions

Although China’s experience in integrating the malaria POR and COVID-19 programs may not be directly comparable to the programs in other countries, the Chinese example highlights the feasibility and sustainability of a highly coordinated program integrating malaria elimination into a COVID-19 response program. It also demonstrates the importance of a sound public health infrastructure with adequate, trained field staff for screening, testing, contact tracing, and providing health education, all of which are crucial for the success of both malaria POR programs and the effective control of COVID-19 in China. Finally, the response to the COVID-19 pandemic must be considered an opportunity to strengthen public health capacity (testing, tracing and isolation) and health system capacity (treatment facilities, medical equipment, and health care workforce), which are crucial for preparedness to control future emerging and reemerging infectious diseases.

## Supplementary Information

**Additional file 1: Figure S1. **The luggage card on which malaria-related online social media QR codes were printed (left). Health staff explain the use of the social medial QR codes to the returnees (right) (The Chinese phrase on the banner in the right photo reads: “Welcome Home”). **Figure S2.** Screening for malaria COVID-19 quarantine stations in Yangzhou, Jiangsu Province, China. **Table S1.** Summary of the focus group discussion questions. **Table S2.** List of the 13 quarantine stations, designated hospitals, and number of the isolated travelers. 

## Data Availability

The data are not publicly available because they contain information that could compromise research participant privacy and consent, but are available from the corresponding author upon reasonable request.
