# Supplementary material for: Implementation and challenges to preventing the re-establishment of malaria in China in the COVID-19 era
Source: Global Health. 2022 Jun 21;18:64. doi: 10.1186/s12992-022-00858-w (PMC9210339; doi:10.1186/s12992-022-00858-w)
Supplement: Supplementary file 1 — Additional file 1: Figure S1. The luggage card on which malaria-related online social media QR codes were printed (left). Health staff explain the use of the social medial QR codes to the returnees (right) (The Chinese phrase on the banner in the right photo reads: “Welcome Home”). Figure S2. Screening for malaria COVID-19 quarantine stations in Yangzhou, Jiangsu Province, China. Table S1. Summary of the focus group discussion questions. Table S2. List of the 13 quarantine stations, designated hospitals, and number of the isolated travelers. [file 12992_2022_858_MOESM1_ESM.docx]

Supplementary materials


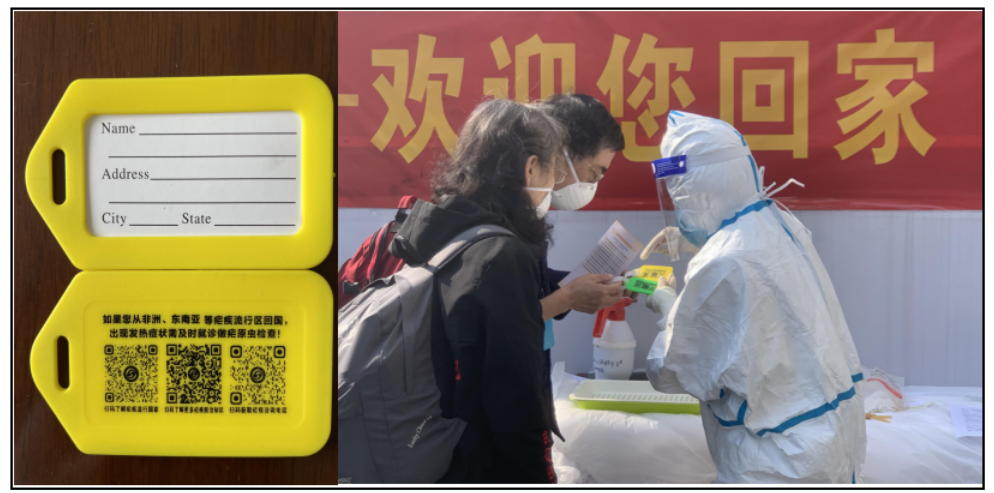


Figure 1. The luggage card on which malaria-related online social media QR codes were printed (left). Health staff explain the use of the social media QR codes to the returnees (right) (The Chinese phrase on the banner in the right photo reads “Welcome Home”).


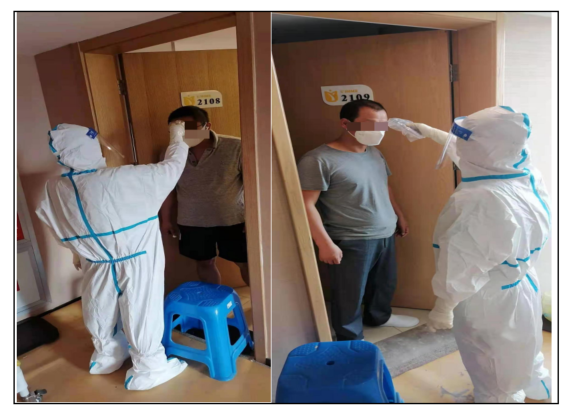


Figure 2. Screening for malaria in COVID-19 quarantine stations in Yangzhou, Jiangsu Province, China

Table 1. Summary of focus group discussion questions

| No. | Summary of the focus group discussion questions |
| --- | --- |
| 1 | Please introduce how you implemented the integrated POR program for malaria and COVID-19 during your work. |
| 2 | In your opinion, what are operational challenges associated with the integrated POR program for malaria and Covid-19? Why? |
| 3 | Among the operational challenges that you have identified, which ones are particularly critical, and do you have any solutions to cope with the challenges? |
| 4 | Have you already implemented interventions to respond these challenges? If yes, how? If no, why not? |
| 5 | Did you learn any lessons from the implementation of the integrated POR program for malaria and Covid-19? If yes, what? |

Table 2. List of the 13 quarantine stations, designated hospitals and number of isolated travelers

| No. | Quarantine stations | Designated hospitals | Number of isolated travelers returning from abroad | Number of isolated travelers returning from malaria-endemic countries | Number of distributed health educational booklets and materials on malaria |
| --- | --- | --- | --- | --- | --- |
| 1 | Lifeng Hotel | Wantou Hospital | 103 | 48 | 103 |
| 2 | Hanting Hotel | Yangzhou City 3^rd^ Hospital | 1568 | 1568 | 2842 |
| 3 | Junyi Hotel | Xian’nv Health Community Center | 129 | 25 | 500 |
| 4 | Gelinhaotai Hotel | Southern City Health Community Center | 371 | 40 | 500 |
| 5 | Hanting Hotel | Xieqiao Health Community Center | 156 | 51 | 500 |
| 6 | Shangyi Hotel | Xian’nv Health Community Center | 341 | 43 | 500 |
| 7 | Hanting Hotel in Development District | Yangzhou University-affiliated hospital | 138 | 21 | 820 |
| 8 | Yiyuan Hotel | Shieryu Health Center in Yizheng | 1477 | 1477 | 6200 |
| 9 | Liming Hotel | Yizheng city hospital | 1886 | 1886 | 7800 |
| 10 | Gelinhaotai Hotel (Nanxiao) | Gaoyou city People’s Hospital | 79 | 32 | 261 |
| 11 | Gelinlianmen Hotel | Baoying county hospital | 185 | 36 | 1800 |
| 12 | Hugang Hotel | Baoying county hospital | 103 | 20 | 1200 |
| 13 | Mingyue Lake Hotel | Yangzhou City 3^rd^ Hospital | 2411 | 315 | 3000 |
| Total |  |  | 8947 | 5562 | 26026 |
